# Reflections on the JCAG editorial fellowship

**DOI:** 10.1093/jcag/gwaf028

**Published:** 2025-11-06

**Authors:** Sama Anvari

**Affiliations:** Division of Gastroenterology, Department of Medicine, McMaster University, Hamilton, ON L8L4L8, United States

For most Canadian medical trainees, the inner workings of an academic journal remain a “black box.” While most are familiar with conducting and publishing research, the process of moving a research article from submission to publication remains enigmatic. This presents a conundrum both for trainees who hope to one day pursue an academic career and for existing editorial board members who may not have a clear avenue to pass their knowledge and experience onto the next generation of clinicians. The *Journal of the Canadian Association of Gastroenterology* (JCAG) editorial fellowship presents an answer to both problems, providing a Canadian Association of Gastroenterology (CAG) trainee with a glimpse into the world of academic publishing while also fostering mentorship and professional development.

I had the privilege of serving as a JCAG Editorial Fellow in my final year of gastroenterology fellowship and found it to be an immensely valuable experience. In this role, I participated in meetings with the publisher, interacted with members of the editorial board, and reviewed manuscripts under the expert guidance of associate editors. Having submitted my fair share of manuscripts to various journals over the years, it was an eye-opening experience to be on the other side of the curtain and provide my feedback on submitted works. I also learned from reviewing the feedback given by other reviewers and comparing it to my own, gaining new perspectives on the nuances of study design and execution. Before the fellowship, I had had the opportunity to peer review a few research works; however, I was neither given a clear framework nor feedback on how to do so effectively. With the support of Dr Bessissow, my faculty mentor, I learned how to effectively critique an article while also providing authors with actionable suggestions. Throughout this process, I was thoroughly impressed by my fellow reviewers’ dedication to providing thoughtful, constructive feedback on submitted works. It was also a humbling and inspiring experience to glimpse some of the excellent research that is currently being performed by colleagues in gastroenterology. Finally, this role gave me a renewed appreciation for the process of research, as well as a better understanding of what elements make up a high-quality submission to an academic journal. I learned the importance of asking the question “Why does this research matter?,” an insight that will apply to my own manuscripts moving forward. As an aspiring academic clinician, I can say without a doubt that this experience served as an invaluable complement to my clinical training in gastroenterology. It made me both a better writer and scientist, while also highlighting many avenues for future growth.

Beyond manuscript review, I also had the opportunity to learn more about the business of medical publishing and sit in on planning meetings with JCAG’s publisher. As physicians, we rarely have the opportunity to appreciate the strategic and operational realities of running a successful journal. During my time as a editorial fellow, JCAG received its first Impact Factor, a huge success and reflection of all the time and effort poured into the journal. It also faced challenges such as attracting reviewers and changes in publishing personnel, as well as a rising awareness of the use of AI in publishing. This experience highlighted that sharing high-quality research is just one of many facets of running an academic journal, a learning point that was highly valuable. As I move into an associate editor position at JCAG, I look forward to seeing how the face of publishing continues to change and how the responsibilities of the editorial board will evolve with it.

Finally, my favourite aspect of the fellowship was the opportunity to participate in educational opportunities led by JCAG. Alongside JCAG’s Editor in Chief, Dr Eric Benchimol, I presented a workshop on peer-reviewing manuscripts to other gastroenterology fellows at the Gastroenterology Residents-in-Training course. I also had the privilege of serving as a panel member at the annual CDDW JCAG Symposium. Both experiences were fantastic learning opportunities to gain presentation skills, and I truly appreciate Dr Benchimol’s support and expertise.

My original goal in applying for the JCAG Editorial Fellowship was to develop as a clinician, researcher, and educator. I can say without a doubt that the fellowship exceeded my expectations. The critical thinking and problem-solving skills I gained through this experience have shaped my approach to both critically appraising research and adapting it to clinical use. I look forward to putting them to use as I complete my fellowship in Advanced Inflammatory Bowel Disease and move into the next chapter of my career. I am truly grateful for the mentorship, guidance, and support of Dr Benchimol and Dr Bessissow, as well as for the opportunity to meet incredible gastroenterologists from across the country. I look forward to continuing at JCAG and seeing the continued evolution of the program, which will undoubtedly benefit future trainees for years to come.

## Data Availability

There are no data associated with this editorial article.

